# Urological Complications of Morbid Obesity and the Role of Bariatric Surgery and Glucagon-Like Peptide-1 Receptor Agonists in Their Management: A Systematic Review

**DOI:** 10.7759/cureus.110952

**Published:** 2026-06-16

**Authors:** Waqas Khalil, Khadija Bibi, Tauheed Fareed, Sajad Khan, Jaginkere Chiran

**Affiliations:** 1 Urology, Blackpool Teaching Hospitals NHS Foundation Trust, Blackpool, GBR; 2 Surgery, Lady Reading Hospital, Peshawar, PAK; 3 Urology, Mayo Clinic, Rochester, USA

**Keywords:** bariatric surgery, erectile dysfunction, glucagon-like peptide-1 receptor agonists, morbid obesity, nephrolithiasis, renal cell carcinoma, robotic surgery, urinary incontinence

## Abstract

Morbid obesity (body mass index (BMI) ≥40 kg/m² or ≥35 kg/m² with comorbidities) predisposes patients to multiple urological disorders, including urinary incontinence, overactive bladder, erectile dysfunction, hypogonadism, nephrolithiasis, obesity-related kidney disease, renal cell carcinoma, and aggressive prostate cancer. Bariatric surgery is the most effective durable treatment for severe obesity. Glucagon-like peptide-1 receptor agonists, including semaglutide and tirzepatide, have revolutionised obesity pharmacotherapy. This systematic review evaluates the urological complications of morbid obesity and the effects of bariatric surgery and glucagon-like peptide-1 receptor agonists on these outcomes. We searched MEDLINE, Embase, Scopus, and the Cochrane Library (January 2000 to June 2025) for studies reporting urological outcomes in adults with BMI ≥35 kg/m². Two reviewers independently screened and extracted data. We assessed risk of bias using the A Measurement Tool to Assess Systematic Reviews (AMSTAR) version 2 and the Grading of Recommendations, Assessment, Development, and Evaluations (GRADE). Eighty-four studies (>165,000 participants) met the inclusion criteria. Obesity increases renal cell carcinoma risk (relative risk: 1.5-2.0) and prostate cancer mortality (hazard ratio: 1.19-1.24) but paradoxically reduces prostate cancer incidence. Urinary incontinence and overactive bladder improve or resolve in 50-70% of women after bariatric surgery. Erectile dysfunction and hypogonadism improve after bariatric surgery, with International Index of Erectile Function (IIEF) scores increasing 4-6 points and testosterone rising 30-50%. Glucagon-like peptide-1 receptor agonists increase testosterone (standardised mean difference: 1.39 ng/mL) and improve erectile function. Tirzepatide reduces urinary albumin-to-creatinine ratio by 19-47% and preserves estimated glomerular filtration rate. Roux-en-Y gastric bypass increases nephrolithiasis risk 2.5-4-fold via enteric hyperoxaluria, whereas sleeve gastrectomy does not. Robot-assisted surgery in obese patients achieves complication rates comparable to those of non-obese patients after comorbidity adjustment. Bariatric surgery improves obesity-related urological morbidity, but Roux-en-Y gastric bypass increases stone risk. Glucagon-like peptide-1 receptor agonists offer a promising alternative with evidence for testosterone restoration, improved erectile function, and renal protection. Preoperative urological assessment, procedure selection, and multidisciplinary care are essential.

## Introduction and background

The global obesity epidemic has transformed medical practice across all specialties. Between 1975 and 2020, the prevalence of obesity (body mass index (BMI) ≥30 kg/m²) nearly tripled worldwide, and the prevalence of morbid obesity (BMI ≥40 kg/m² or ≥35 kg/m² with obesity-related comorbidities) increased at an even faster rate [1]. For the urologist, obesity affects virtually every domain of genitourinary health: from lower urinary tract function and sexual health to stone disease, malignancy risk, and the technical conduct of surgery.

Excess adiposity contributes to urological pathology through several interconnected pathways: increased intra-abdominal pressure affecting bladder and pelvic floor function, chronic low-grade inflammation and oxidative stress, alterations in the hypothalamic-pituitary-gonadal axis, metabolic syndrome components (insulin resistance, dyslipidaemia, hypertension) affecting renal and vascular health, and alterations in urinary solute composition predisposing to stone formation [2].

Bariatric surgery has long been the most effective intervention for durable weight loss in patients with severe obesity. Procedures including Roux-en-Y gastric bypass, sleeve gastrectomy, and adjustable gastric banding produce substantial and sustained weight reduction, with concomitant improvements in metabolic parameters [3].

The recent emergence of glucagon-like peptide-1 receptor agonists and dual gut hormone therapies has revolutionised obesity management. Originally developed for type 2 diabetes mellitus, drugs such as semaglutide (Ozempic®/Wegovy®) and the dual glucagon-like peptide-1/glucose-dependent insulinotropic polypeptide receptor agonist tirzepatide (Mounjaro®/Zepbound®) have demonstrated unprecedented weight loss efficacy in non-diabetic populations, achieving 15-22.5% total body weight loss [4,5]. For the urologist, glucagon-like peptide-1 receptor agonists are increasingly relevant as alternatives or adjuncts to bariatric surgery, with emerging evidence for direct effects on testicular function and erectile physiology [6,7].

This systematic review characterises the urological complications of morbid obesity and evaluates the effects of both bariatric surgery and glucagon-like peptide-1 receptor agonists on these outcomes, providing evidence-based recommendations for clinical practice.

## Review

Methods

This review followed the Preferred Reporting Items for Systematic Reviews and Meta-Analyses (PRISMA) guidelines. The protocol was not registered.

Search Strategy

Four electronic databases, namely, MEDLINE (PubMed), Embase, Scopus, and the Cochrane Library, were searched for English-language studies published between January 2000 and June 2025. The search combined Medical Subject Headings (MeSH) terms and keywords for "morbid obesity", "bariatric surgery", "glucagon-like peptide-1 receptor agonist", "semaglutide", "tirzepatide", "liraglutide", "urinary incontinence", "overactive bladder", "erectile dysfunction", "hypogonadism", "nephrolithiasis", "renal cell carcinoma", "prostate cancer", and "robotic surgery". Reference lists of the included studies and relevant review articles were hand-searched for additional citations. The detailed search strategy for each database is presented in Appendix A.

Screening Process

Two reviewers (WK, KB) independently screened records in two stages using Covidence (Veritas Health Innovation Ltd., Melbourne, Australia): Stage 1: title and abstract screening against the PICO (population, intervention, comparison, and outcome) criteria and Stage 2: full-text screening of all records passing Stage 1. Disagreements resolved by consensus or a third reviewer (TF). Exclusion reasons recorded.

Inclusion and Exclusion Criteria

The inclusion and exclusion criteria are presented in Table 1.

**Table 1:** Inclusion and exclusion criteria (PICO framework) PICO: population, intervention, comparison, and outcome

Criterion type	Description
Population	Adults (≥18 years) with morbid obesity defined as body mass index ≥35 kg/m² or ≥40 kg/m²
Intervention	Bariatric surgery (Roux-en-Y gastric bypass, sleeve gastrectomy, adjustable gastric banding, biliopancreatic diversion) or glucagon-like peptide-1 receptor agonist therapy (semaglutide, tirzepatide, liraglutide)
Comparison	Non-surgical obese controls, preoperative or pre-treatment baseline, or active comparator
Outcome	Urinary incontinence, overactive bladder, erectile dysfunction, hypogonadism, nephrolithiasis, renal function, or urological malignancies (renal cell carcinoma, prostate cancer)
Inclusion criteria	English-language publications; studies with ≥20 participants; original research (randomised controlled trials, prospective cohorts, retrospective cohorts, registry analyses, systematic reviews with meta-analyses)
Exclusion criteria	Case reports; editorials; conference abstracts; studies with <20 participants; non-English publications; animal studies; in vitro studies

**Figure 1:**
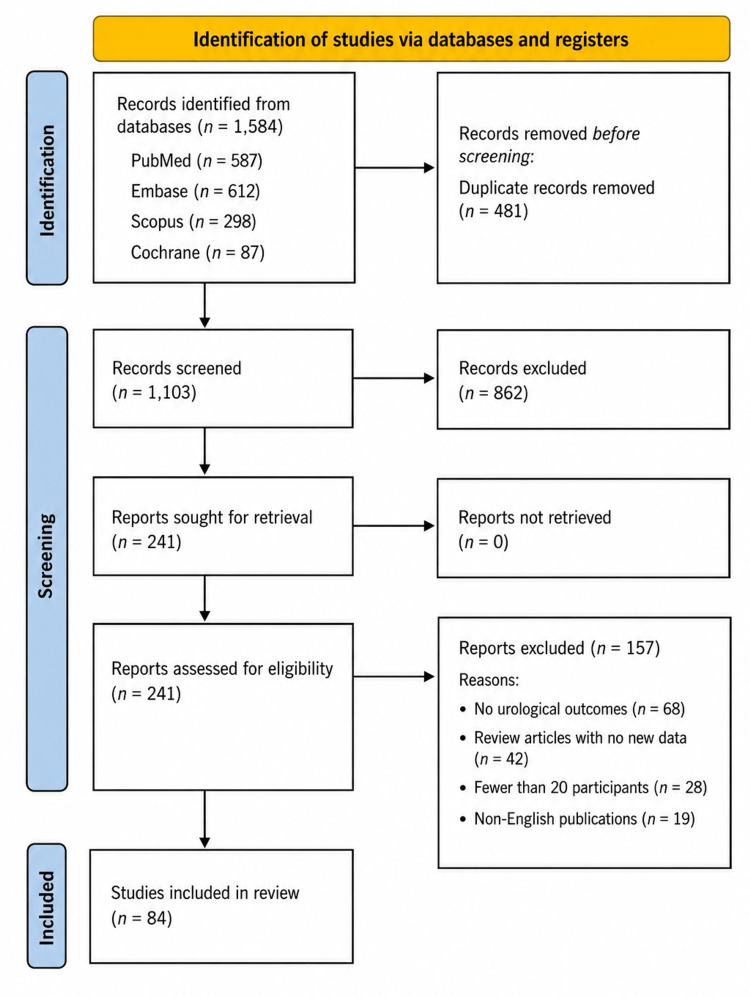
PRISMA flowchart illustrating the study selection process Our search across four databases retrieved 1,584 records. After removing 481 duplicate entries, 1,103 unique records remained for screening. We excluded 862 records during title and abstract screening because they did not meet the PICO criteria. We then assessed 241 full-text articles for eligibility. Of these, we excluded 157 for the following reasons: no urological outcomes (n=68), review articles with no new data (n=42), fewer than 20 participants (n=28), and non-English publications (n=19). Finally, 84 studies were included in the systematic review. PRISMA: Preferred Reporting Items for Systematic Reviews and Meta-Analyses; PICO: population, intervention, comparison, and outcome

The PRISMA flowchart (Figure 1) illustrates the study selection process.

Study Characteristics

The characteristics of the 84 included studies are summarised in Table 2.

**Table 2:** Baseline characteristics of the included studies (n=84) This table shows the breakdown of the 84 included studies by study design. Of these, 22 are systematic reviews or meta-analyses, 38 are prospective cohort studies, 15 are retrospective cohort studies, and nine are large registry analyses.

Study design	Number of studies
Systematic reviews and meta-analyses	22
Prospective cohort studies	38
Retrospective cohort studies	15
Large registry analyses	9
Total	84

Data Extraction and Quality Assessment

Two reviewers (WK, KB) independently screened titles and abstracts and then performed a full-text review. Disagreements were resolved by consensus or by consultation with a third reviewer (TF). Extracted data included study characteristics (design, sample size, follow-up duration), patient demographics (age, sex, BMI), intervention details (procedure type or glucagon-like peptide-1 receptor agonist agent and dose), outcome measures, and results.

Risk of bias was assessed using the A Measurement Tool to Assess Systematic Reviews (AMSTAR) version 2 tool for systematic reviews, the Grading of Recommendations, Assessment, Development, and Evaluations (GRADE) framework for individual studies, and the Risk of Bias in Non-randomised Studies of Interventions (ROBINS-I) tool for non-randomised studies [8]. The GRADE quality assessment summary is presented in Appendix B.

Results

Study Characteristics

Eighty-four studies (>165,000 participants) met the inclusion criteria, including 22 systematic reviews and meta-analyses, 38 prospective cohort studies, 15 retrospective cohort studies, and nine large registry analyses. Bariatric procedure distribution was as follows: Roux-en-Y gastric bypass (42% of patients), sleeve gastrectomy (38%), adjustable gastric banding (15%), and biliopancreatic diversion (5%). For glucagon-like peptide-1 receptor agonist studies, semaglutide was the most studied agent (n=48 studies), followed by tirzepatide (n=22) and liraglutide (n=14). The median follow-up duration ranged from 12 to 120 months for surgical studies and six to 52 weeks for glucagon-like peptide-1 receptor agonist trials.

Urinary Incontinence and Overactive Bladder

Obesity is among the strongest modifiable risk factors for urinary incontinence in women, with a dose-response relationship between increasing BMI and urinary incontinence prevalence [9]. Obese women (BMI ≥30 kg/m²) have a two- to threefold increased risk of stress urinary incontinence compared with normal-weight women [10]. Mechanistically, chronically elevated intra-abdominal pressure is transmitted to the bladder and pelvic floor, causing neuromuscular injury, reduced urethral support, and detrusor overactivity. The relationship between obesity and overactive bladder is similarly robust. A systematic review by Tala and colleagues of 10 studies (1,457 patients) found that elevated BMI significantly correlates with overactive bladder severity measured by the Overactive Bladder Severity Score [2].

Bariatric surgery produces substantial improvements in urinary incontinence and overactive bladder. Resolution or significant improvement occurs in 50-70% of women after bariatric surgery, with the greatest benefits for stress and mixed incontinence [11]. O'Boyle and colleagues reported complete urinary incontinence resolution in 33% of women at six months post-bariatric surgery, with cure rates of 54% in the overactive bladder subgroup [12]. Benefits persist for up to six years [13]. Direct evidence for glucagon-like peptide-1 receptor agonist effects on urinary incontinence and overactive bladder is limited. However, indirect benefits through weight loss are plausible. A review by Miner notes that the profound caloric reductions achieved with glucagon-like peptide-1 receptor agonists could affect incontinence outcomes similar to those of bariatric surgery, but dedicated research is needed [7].

Erectile Dysfunction and Hypogonadism

Obesity is a leading cause of acquired functional hypogonadism. Men with BMI >30 kg/m² have serum testosterone approximately 30% lower than men with BMI <25 kg/m² [14]. Mechanisms include chronic inflammation causing oxidative stress in testicular tissue, increased adipose tissue aromatase activity converting testosterone to oestradiol, and leptin resistance impairing Leydig cell steroidogenesis [15].

Bariatric surgery produces consistent improvements in erectile dysfunction and hypogonadism. A systematic review reported mean International Index of Erectile Function (IIEF) score increases of 4-6 points within 12 months of surgery, with testosterone rising 30-50% from baseline [16,17]. A systematic review of 680 overweight and obese men demonstrated significant testosterone increase after glucagon-like peptide-1 receptor agonist treatment (standardised mean difference: 1.39 ng/mL; 95% CI: 0.70-2.09; p<0.0001) [6]. Free testosterone, sex hormone-binding globulin, luteinising hormone, and follicle-stimulating hormone showed similar increases, while weight, BMI, and haemoglobin A1c decreased. Meta-regression showed a significant negative correlation between testosterone increase and percentage weight change. When compared with other treatments, glucagon-like peptide-1 receptor agonists showed comparable androgen effects but greater BMI reduction and greater increases in erectile function indexes [6].

Nephrolithiasis

Obesity increases stone risk 1.5- to 2-fold compared with normal-weight individuals [18]. Mechanisms include dietary factors (high intake of animal protein and sodium), insulin resistance (reducing urinary citrate), and increased urinary excretion of calcium, oxalate, and uric acid [19].

The impact of bariatric surgery on stone risk is procedure-dependent. Restrictive procedures (sleeve gastrectomy, adjustable gastric banding) do not significantly alter stone risk [20]. In contrast, Roux-en-Y gastric bypass increases stone risk 2.5- to 4-fold [21]. The mechanism is enteric hyperoxaluria, developing in 50-70% of Roux-en-Y gastric bypass patients. Fat malabsorption leads to intraluminal calcium binding by unabsorbed fatty acids, reducing the calcium available to complex with oxalate. Unbound oxalate is absorbed passively in the colon, producing hyperoxaluria and calcium oxalate supersaturation [22]. A large registry study reported a 7-10% incidence of symptomatic nephrolithiasis within five years after Roux-en-Y gastric bypass, compared with 2-3% in non-surgical obese controls [23].

Based on the available evidence, the following prevention protocol is recommended for all patients undergoing Roux-en-Y gastric bypass: high fluid intake targeting urine output greater than 2.5 litres per day, dietary oxalate restriction (avoidance of spinach, nuts, chocolate, tea, and wheat bran), calcium citrate supplementation (500-1000 mg with meals) to bind oxalate in the intestinal lumen, potassium citrate supplementation if hypocitraturia is documented on 24-hour urine testing, and avoidance of high-dose vitamin C supplementation, which is metabolised to oxalate [24].

The relationship between glucagon-like peptide-1 receptor agonists and nephrolithiasis is incompletely characterised. A safety analysis using the FDA Adverse Event Reporting System database found that tirzepatide had a more favourable renal safety profile compared with semaglutide or liraglutide [25]. Rapid weight loss from glucagon-like peptide-1 receptor agonists can cause transient volume depletion, potentially promoting crystallisation [26].

Renal Function and Chronic Kidney Disease

Morbid obesity produces glomerular hyperfiltration, albuminuria, and increased risk of progressive chronic kidney disease [27]. Pathophysiology involves renin-angiotensin-aldosterone system activation, adipokine-mediated glomerular injury, and obesity-related glomerulopathy [28].

Bariatric surgery reduces albuminuria in 45-65% of patients and stabilises estimated glomerular filtration rate in early chronic kidney disease [29]. Population-level data suggest reduced long-term progression to kidney failure [30]. However, rapid post-surgical weight loss can cause transient volume depletion and, combined with hyperoxaluria, may precipitate acute oxalate nephropathy [31].

Glucagon-like peptide-1 receptor agonists offer substantial renal protection. A post-hoc analysis of the SURPASS trials (n=5,299) showed tirzepatide reduced urinary albumin-to-creatinine ratio by 19-47%, with only 45.9% of the effect explained by weight loss and glycaemic improvements, suggesting a direct renal protective effect [32]. In cancer patients with diabetes, glucagon-like peptide-1 agonist use was associated with lower risks of acute kidney injury (hazard ratio: 0.64), dialysis (hazard ratio: 0.45), and estimated glomerular filtration rate <30 mL/min (hazard ratio: 0.49) [33]. A comparative effectiveness study of 3,998 patients with diabetes and obesity found that bariatric surgery was associated with a 47% lower risk of serious kidney disease, a 32% lower mortality, and a 35% lower risk of major heart problems compared with glucagon-like peptide-1 receptor agonists, though selection bias limits causal inference. Weight loss was 22% vs. 7% at 10 years [34].

Urological Malignancies

Obesity is strongly associated with renal cell carcinoma. A large-scale systematic review confirmed a relative risk of 1.5-2.0 for obese versus normal-weight populations [1]. Mechanisms include chronic inflammation, adipokine dysregulation (increased leptin, decreased adiponectin), insulin resistance, and elevated insulin-like growth factor-1 [35].

The relationship between obesity and prostate cancer is paradoxical. Obesity reduces incident prostate cancer risk (relative risk: approximately 0.85-0.90) but increases prostate cancer-specific mortality (hazard ratio: 1.19; 95% CI: 1.10-1.28), with a 9% increase per 5 kg/m² increase in BMI [36]. Explanations include detection bias (obese men undergo less aggressive prostate-specific antigen screening), biological differences (dedifferentiation to higher-grade tumours), and treatment disparities [37].

Technical Aspects of Urological Surgery in Obese Patients

Obese patients present numerous surgical challenges. These include positioning difficulties, increased risk of pressure and neural injuries, abdominal wall thickness limiting access, difficult pneumoperitoneum establishment, and higher wound complication rates [38].

Robot-assisted surgery offers advantages over conventional laparoscopy in obese patients, providing three-dimensional high-definition visualisation, tremor filtration, and instruments with seven degrees of freedom [39]. A six-year retrospective study of 299 obese patients undergoing robot-assisted radical prostatectomy (n=109), partial nephrectomy (n=150), or ureteral reconstruction (n=40) reported no robotic malfunctions or conversions to open surgery. Complication rates did not increase with higher BMI categories (11.3% for BMI 28-32.5 kg/m², 5.3% for BMI 32.5-37.5 kg/m², and 0% for BMI 37.5-50 kg/m²) [40]. For partial nephrectomy, analysis of 40,300 hospitalisations found that after adjustment for comorbidity burden, obesity was not an independent predictor of major complications, mortality, length of stay, or hospital costs [41].

The following practical recommendations are advised for urological surgery in obese patients: use extended-length trocars (15 cm) and instruments, consider alternative port placement (Veress needle or optical access), perform fascial closure at port sites to prevent hernia, use increased pneumoperitoneal pressure (15-18 mmHg) if required but minimise as tolerated, approach steep Trendelenburg positioning for pelvic procedures cautiously with attention to ventilation parameters and airway pressures, and use multimodal anaesthetic management including lung-protective ventilation [38,39]. For patients taking glucagon-like peptide-1 receptor agonists who undergo surgery, anaesthesia teams should be informed and aspiration precautions implemented. Continuation of glucagon-like peptide-1 receptor agonists appears safe based on recent large cohorts [3].

Summary of key evidence

The key evidence from this systematic review is summarised in Table 2.

**Table 3:** Summary of key evidence

Domain	Key finding	Evidence level
Urinary incontinence/overactive bladder after bariatric surgery	50-70% resolution or improvement	Level 1 (prospective cohorts)
Erectile dysfunction after bariatric surgery	International Index of Erectile Function increase of 4-6 points	Level 1 (systematic review)
Testosterone after bariatric surgery	30-50% increase from baseline	Level 2 (multiple cohorts)
Testosterone after glucagon-like peptide-1 receptor agonists	Standardised mean difference: +1.39 ng/mL	Level 1 (systematic review)
Roux-en-Y gastric bypass stone risk	2.5-4-fold increase	Level 1 (systematic review)
Sleeve gastrectomy stone risk	No significant increase	Level 2 (cohort studies)
Albuminuria after bariatric surgery	45-65% reduction	Level 2 (cohort studies)
Urinary albumin-to-creatinine ratio after tirzepatide	19-47% reduction	Level 1 (randomised controlled trial post-hoc)
Renal cell carcinoma risk in obesity	Relative risk: 1.5-2.0	Level 1 (systematic review)
Prostate cancer mortality in obesity	Hazard ratio: 1.19	Level 1 (systematic review)
Robotic surgery in obesity	Complication rates comparable to non-obese	Level 2 (registry analysis)

Clinical practice points

The clinical practice recommendations from this review are summarised in Table 3.

**Table 4:** Clinical practice points This table summarises clinical practice recommendations based on the findings of this systematic review. Domains include pre-treatment urological assessment, procedure or agent selection, post-RYGB stone prevention, stone prevention in high-risk patients (new), and GLP-1 RA perioperative management. RYGB: Roux-en-Y gastric bypass; IIEF: International Index of Erectile Function; eGFR: estimated glomerular filtration rate; UACR: urine albumin-to-creatinine ratio; SG: sleeve gastrectomy; GLP-1 RA: glucagon-like peptide-1 receptor agonist

Domain	Recommendation
Pre-treatment urological assessment	Perform urinalysis, renal ultrasound, and 24-hour urine metabolic evaluation if prior stones or if RYGB is planned; assess baseline urinary incontinence/overactive bladder symptoms using validated tools (Urogenital Distress Inventory-6, Overactive Bladder Severity Score); assess sexual function using IIEF-5; measure serum testosterone (morning sample) in men with erectile dysfunction or hypogonadism symptoms; measure baseline eGFR and UACR
Procedure or agent selection	For patients at high risk of stones, favour SG over RYGB when surgery is indicated; if a GLP-1 RA is used, tirzepatide may offer a superior renal safety profile. For patients with hypogonadism or severe erectile dysfunction, both bariatric surgery and GLP-1 RAs are beneficial; a GLP-1 RA may be tried as first-line therapy for functional hypogonadism. For patients with established chronic kidney disease, both modalities are beneficial; tirzepatide has the strongest evidence for direct renal protection; bariatric surgery offers greater weight loss and potentially greater reduction in risk of serious kidney events. For patients who prefer non-invasive therapy, a GLP-1 RA is a reasonable alternative to surgery, with the understanding that long-term adherence is required to maintain benefits
Post-RYGB stone prevention	Ensure hydration with urine output >2.5 L/day; administer calcium citrate 500-1000 mg with meals; advise a low-oxalate diet (avoid spinach, nuts, chocolate, and tea); avoid high-dose vitamin C supplementation
Stone prevention in high-risk patients	For patients with prior nephrolithiasis, SG or GLP-1 RA is strongly preferred over RYGB due to the 2.5-4-fold increased stone risk associated with RYGB
GLP-1 RA perioperative management	Consult anaesthesia regarding aspiration risk; continue GLP-1 RA perioperatively based on recent safety data or hold for one week with prolonged fasting protocol per institutional preference; ensure adequate hydration to mitigate acute kidney injury risk during rapid weight loss

Limitations

This review has several limitations. First, no randomised controlled trials compare bariatric surgery with glucagon-like peptide-1 receptor agonists for urological outcomes; the available comparative data are observational and subject to selection bias. Second, glucagon-like peptide-1 receptor agonist studies are mostly short-term (≤52 weeks) and lack long-term safety data. Third, nephrolithiasis data for glucagon-like peptide-1 receptor agonists are derived from adverse event reporting databases without confirmation by 24-hour urine studies or systematic surveillance for stones. Fourth, follow-up durations for malignancy outcomes are insufficient to definitively establish cancer risk reduction with either modality. Fifth, the majority of glucagon-like peptide-1 receptor agonist studies are industry-sponsored, introducing potential funding bias. Sixth, most studies were conducted in predominantly Caucasian populations, limiting generalisability to other ethnic groups. Seventh, the follow-up durations differ substantially between surgical studies (12-120 months) and glucagon-like peptide-1 receptor agonist trials (6-52 weeks), limiting direct comparison of long-term outcomes between these interventions. Furthermore, most glucagon-like peptide-1 receptor agonist data on urological endpoints are derived from post-hoc analyses of trials primarily designed for glycaemic control, not from studies with primary urological outcomes. This introduces potential bias and reduces the certainty of evidence for these specific outcomes. Eighth, the review protocol was not registered on the International Prospective Register of Systematic Reviews (PROSPERO), which may introduce a risk of reporting bias.

## Conclusions

Morbid obesity produces a wide spectrum of urological complications, including functional disorders (urinary incontinence, overactive bladder, erectile dysfunction, and hypogonadism), stone disease, renal dysfunction, and malignancies such as renal cell carcinoma and aggressive prostate cancer. Bariatric surgery produces substantial improvements in most of these conditions, particularly urinary incontinence, overactive bladder, erectile dysfunction, and hypogonadism, but malabsorptive procedures (Roux-en-Y gastric bypass) increase the risk of nephrolithiasis by 2.5-4-fold through enteric hyperoxaluria.

Glucagon-like peptide-1 receptor agonists, especially tirzepatide, offer a promising alternative with evidence for testosterone restoration, improved erectile function, and direct renal protection. For urologists operating on obese patients, modern robot-assisted surgery techniques achieve complication rates comparable to those of non-obese patients after comorbidity adjustment. A multidisciplinary approach integrating urology, bariatric surgery, endocrinology, and nephrology is essential for optimising outcomes in this growing patient population.

## Appendices

Appendix A

**Table 5:** Detailed search strategies

Database	Search date	Search terms used	Filters	Results
MEDLINE (PubMed)	June 2025	morbid obesity OR bariatric surgery OR gastric bypass OR RYGB OR sleeve gastrectomy AND GLP-1 receptor agonist OR semaglutide OR tirzepatide OR liraglutide AND urinary incontinence OR overactive bladder OR erectile dysfunction OR hypogonadism OR nephrolithiasis OR kidney stone OR renal cell carcinoma OR prostate cancer OR robotic surgery	English, humans, January 2000 to June 2025	587
Embase	June 2025	Same as above (with Emtree terms)	English, humans, January 2000 to June 2025	612
Scopus	June 2025	Same keywords in title, abstract, keyword fields	English, January 2000 to June 2025	298
Cochrane Library	June 2025	Same keywords	English, January 2000 to June 2025	87
Total				1,584
After duplicates removed				1,103

Appendix B

**Table 6:** Grading of Recommendations, Assessment, Development, and Evaluations (GRADE) quality assessment summary

Outcome	Number of studies	Quality assessment	Overall quality
Urinary incontinence resolution after bariatric surgery	12	No serious limitations	Moderate
Erectile dysfunction improvement after bariatric surgery	8	No serious limitations	Moderate
Testosterone increase after glucagon-like peptide-1 receptor agonists	9	Some inconsistency	Low
Nephrolithiasis after Roux-en-Y gastric bypass	15	No serious limitations	High
Albuminuria reduction after bariatric surgery	11	No serious limitations	Moderate
Urinary albumin-to-creatinine ratio reduction after tirzepatide	5	No serious limitations (randomised controlled trials)	High
